# Viral Infections Boost Prokaryotic Biomass Production and Organic C Cycling in Hadal Trench Sediments

**DOI:** 10.3389/fmicb.2019.01952

**Published:** 2019-08-23

**Authors:** Elisabetta Manea, Antonio Dell’Anno, Eugenio Rastelli, Michael Tangherlini, Takuro Nunoura, Hidetaka Nomaki, Roberto Danovaro, Cinzia Corinaldesi

**Affiliations:** ^1^Department of Life and Environmental Sciences, Polytechnic University of Marche, Ancona, Italy; ^2^Stazione Zoologica Anton Dohrn, Naples, Italy; ^3^Research Center for Bioscience and Nanoscience (CeBN), Japan Agency for Marine-Earth Science and Technology (JAMSTEC), Yokosuka, Japan; ^4^Institute for Extra-Cutting-Edge Science and Technology Avant-Garde Research (X-star), Japan Agency for Marine-Earth Science and Technology (JAMSTEC), Yokosuka, Japan; ^5^Department of Sciences and Engineering of Materials, Environment and Urbanistics, Polytechnic University of Marche, Ancona, Italy

**Keywords:** hadal trenches, viruses, viral lysis, deep-sea ecosystems, benthic prokaryotes

## Abstract

Hadal trenches are among the most remote and least explored ecosystems on Earth and can support high benthic microbial standing stocks and activities. However, information on the role of viruses in such ecosystems and their interactions with prokaryotic hosts is very limited. Here, we investigated activities of benthic viruses and prokaryotes and their interactions in three hadal trenches (Japan, Izu-Ogasawara and Mariana trenches) and in their nearby abyssal sites. Our findings reveal that these hadal trenches, compared with the surrounding abyssal sites, support higher abundances and biomasses of prokaryotes. In addition, the high prokaryotic biomasses of hadal trenches could favor high rates of viral infection and cell lysis, especially in the Japan Trench. Hadal viruses can release large amounts of highly labile and promptly available organic material by inducing cell lysis, which could contribute to sustain benthic prokaryotes and decrease their dependency on the enzymatic digestion of the more refractory fraction of sediment organic matter. Our results suggest that this process can contribute to explain the discrepancy between high prokaryote biomass and apparent low efficiency in the utilization of the sedimentary organic matter in the hadal ecosystems. Concluding, hadal trenches may be characterized by a highly dynamic viral component, which can boost prokaryotic biomass production, thereby profoundly influencing the functioning of these remote and extreme ecosystems.

## Introduction

The deepest areas of the ocean, commonly defined as ultra-abyssal or hadal zones (>6500 m depth), account for ∼1.5% of the global deep-sea floor and cover ∼45% of the oceanic depth range (Belyaev, 1989; Jamieson, 2015). They are represented almost exclusively by trenches, which are habitats characterized, among the others, by very high hydrostatic pressure and low temperature (Lauro and Bartlett, 2008; Nunoura et al., 2015), and, also due to their remoteness, they are among the least explored ecosystems on Earth (Jamieson et al., 2010). To date, 37 deep-sea trenches have been discovered, 28 of which (including the 9 deepest ones) are located in the Pacific Ocean, at depths ranging between 9 and 11 km (Belyaev, 1989; Watling et al., 2013). Previous studies suggested that deep-sea trenches can act as a depocenter of organic matter supporting high benthic biomass and highly active benthic microbial communities thanks to their peculiar topography (Danovaro et al., 2003; Glud et al., 2013; Ichino et al., 2015; Leduc et al., 2015). In the last decades, microbial communities in hadal ecosystems have been increasingly explored and benthic prokaryotic abundances from Pacific hadal trenches have been reported to be in the order of 10^6^–10^8^ cells cm^–3^ of surface sediment (Danovaro et al., 2003; Glud et al., 2013; Wenzhöfer et al., 2016; Nunoura et al., 2018), thus falling in the range of values found in other deep-sea ecosystems (Danovaro et al., 2005; Middelboe et al., 2006; Wei et al., 2010; Corinaldesi et al., 2019). Despite there is evidence that benthic prokaryotes and viruses in the top cm of deep-sea sediments represent 50–80% of the total microbial biomass and 10–30% of the total biomass on Earth (Whitman et al., 1998; Corinaldesi, 2015; Danovaro et al., 2015), their dynamics and diversity in these extreme systems are still largely unknown. Even less information is available dealing with the role of viruses in such ecosystems and their interactions with prokaryotic hosts.

Globally, viruses have been estimated to be at least one order of magnitude more abundant than prokaryotes and positively correlated to them (Suttle, 2005, 2007; Corinaldesi, 2015). Viruses are important players in microbial food webs, being agents of mortality and mediators of carbon and nutrient cycling, thus contributing to the functioning of the trophic webs (Fuhrman, 1999). By killing their hosts, viruses can transform the living biomass into organic detritus, which can then be used again by other microbes stimulating their growth (Suttle, 2007; Corinaldesi et al., 2012). Viral infections in deep-sea sediments are responsible for the abatement of 80% of prokaryotic heterotrophic production, releasing on a global scale ≈0.37–0.63 Gt of carbon per year, essentially contributing to the functioning of the deep-sea ecosystems (Danovaro et al., 2008b). However, benthic viral abundances reported so far from the Izu-Ogasawara and Mariana trenches are relatively low, ranging from 10^6^ to 10^7^ viruses cm^–3^ (Yoshida et al., 2013), leading to hypothesize a potential low impact on their prokaryotic hosts. Besides this hypothesis, the growing evidence of the high environmental heterogeneity of hadal systems (Stewart and Jamieson, 2018), which can lead to a high variability in the contribution of microbes to ecosystem functioning, suggests that virus–host interactions are not predictable without comparative studies on different trench ecosystems.

In the present study, we compared the results obtained from the surface sediments of three Northwest-Pacific hadal trenches: the Japan, Izu-Ogasawara, and Mariana trenches. The Japan Trench (8000 m deep) is the northernmost among the three investigated trenches and is subjected to high sedimentary inputs from both surface waters and land thanks to its proximity to the continent (Lutz et al., 2007; Watling et al., 2013; Turnewitsch et al., 2014). The Izu-Ogasawara Trench (9700 m deep) extends from a mesotrophic area to the northernmost section of the Mariana Trench, which instead is located under oligotrophic surface waters (Wenzhöfer et al., 2016). The Mariana Trench includes the deepest point on Earth (at 11 km depth at the Challenger Deep; Gvirtzman and Stern, 2004; Nakanishi and Hashimoto, 2011). Despite the different productivity of the surface waters overlying the three trenches, the food availability in these systems is driven also by downslope transport of sedimentary material (Turnewitsch et al., 2014). Moreover, cyclonic currents of deep waters flowing northward affect these systems (Mantyla and Reid, 1983; Johnson, 1998; Fujio et al., 2000), which are even subjected to frequent earthquakes (Hashimoto et al., 2009; Kawagucci et al., 2012; Oguri et al., 2013; Gamo and Shitashima, 2018; Kioka et al., 2019) thus favoring occasional gravity-driven sediment slides and further affecting habitat stability.

In this study, we analyzed distribution, activity and interactions between viruses and prokaryotes in the surface sediments at the bottom of the Japan, Izu-Ogasawara, and Mariana trenches, and in nearby abyssal plain sites. We also investigated the potential implications of viral infection and lysis of prokaryotic hosts on the functioning of these extreme systems in order to provide new insights into hadal microbial ecology.

## Materials and Methods

### Study Area and Sample Collection

**FIGURE 1:**
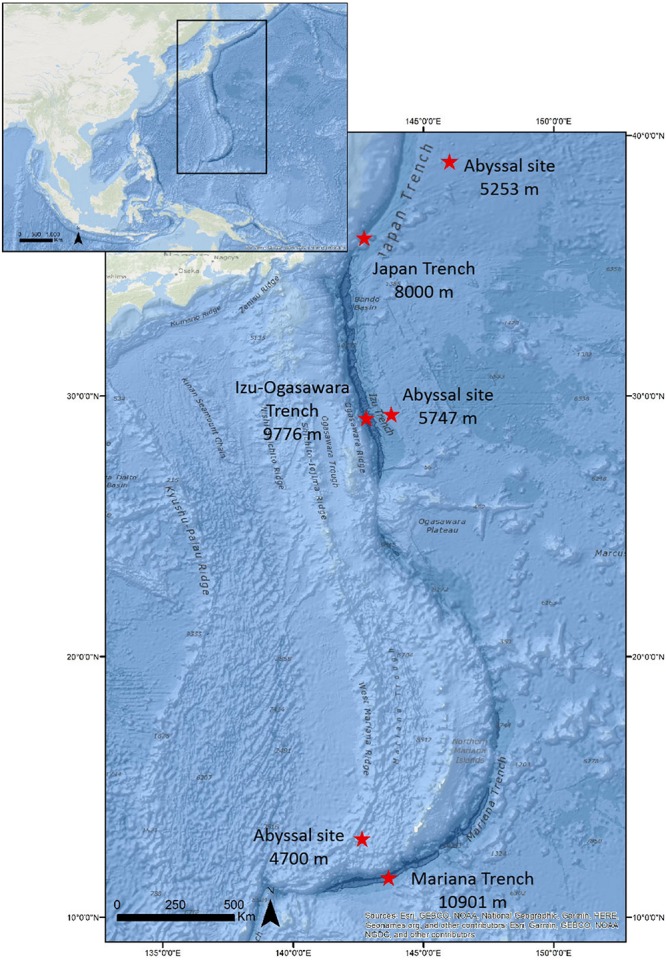
Sampling sites and related depths.

Surface sediment samples were collected from the three Northwest-Pacific hadal trenches (the Japan, Izu-Ogasawara, and Mariana trenches) and also from adjacent abyssal sites (Figure 1). Sediment cores (KR11-11D15 and KR11-11D16) were collected by using a gravity corer with the ROV *ABISMO* at the Izu-Ogasawara Trench (29°09.00′ N, 142°48.12′ E, 9776 m water depth) and at the adjacent abyssal plain on the Pacific plate (29°16.79′ N, 143°46.04′ E, 5747 m water depth), respectively, during the JAMSTEC KR11-11 cruise (R/V *Kairei*) in Dec. 2011 (Figure 1). A sediment core (KR12-19LC2) was also collected at the Japan Trench (36°04.00′ N, 142°44.00′ E, 8000 m water depth) using a 11K lander system (Murashima et al., 2009) during the JAMSTEC KR12-19 cruise (R/V *Kairei*) in December 2012. Sediment cores (6K#1394-R5, R6, and R7) were obtained from the North Pacific abyssal plain (39°00.05′ N, 146°00.13′ E, 5256 m water depth) using a push corer during Shinkai 6500 dive 1394 of the JAMSTEC YK14-06 cruise (R/V *Yokosuka)* in 2014. Finally, during the JAMSTEC KR14-01 cruise (R/V *Kairei)* in January 2014, a sediment core (KR14-01 C1-2) was collected using the 11K lander system from the Challenger Deep of the Mariana Trench (11°22.05′ N, 142°25.45′ E, 10901 m water depth) and an adjacent abyssal sediment cores (KR14-01 E) were obtained using a multiple corer (10°17.98′ N, 142°36.02′ E, 4700 m water depth). Sub-aliquots of the surface 0 to 1 cm depth in sediment cores were used to carry out the analysis of the biochemical composition of the organic matter and microbiological parameters. Immediately after the sediments collection, the samples for the analyses of the biochemical composition of organic matter (i.e., proteins, carbohydrates, lipids, and total phytopigments) were frozen and stored at −20°C. For the analyses of prokaryotic abundances, samples were fixed with buffered 2% formalin and stored at 4°C until further processing on land. Samples for determining viral production rates were processed on board immediately after the collection of the sediments. All analyses were carried out for three replicates.

### Sedimentary Organic Matter

Chlorophyll-*a* and phaeopigments were extracted (12 h at 4°C, in the dark) in 5 ml of 90% acetone, and the extracts were analyzed fluorometrically to estimate chlorophyll-*a*, and after acidification with 0.1 N HCl to estimate the phaeopigments (Danovaro, 2010). The sum of chlorophyll-*a* and phaeopigments concentrations was expressed as total phytopigments (Danovaro, 2010). The concentrations of proteins, carbohydrates, and lipids were measured spectrophotometrically (Danovaro, 2010), and the sum of their concentrations converted into carbon equivalents (by using the conversion factors of 0.49, 0.40, and 0.75 mg C mg^–1^, respectively) was defined as biopolymeric carbon (Danovaro, 2010).

### Prokaryotic Abundance, Cell Size, and Biomass

Total prokaryotic cell counts were performed using the SYBR Green I direct count procedure (Danovaro, 2010). Briefly, the sediment samples were treated by ultrasounds (Branson Sonifier 2200, 60 W) three times for 1 min after addition of 0.2 μm pre-filtered tetrasodium pyrophosphate solution (final concentration, 5 mM), then properly diluted before filtration onto 0.2 μm pore-size Nuclepore black filters (Whatman). Filters were then stained with 20 μl of SYBR Green I (10000 × stock) diluted 1:20 in pre-filtered TE buffer (pH 7.5), with excess stain removed 3 times using 3 ml of Milli-Q water, and mounted onto microscope slides. At least 20 microscope fields and 400 cells per filter were counted randomly using epifluorescence microscopy (EFM) under blue light (Zeiss Axioskop 2MOT, magnification: 1000×; excitation BP 450–490 nm). For the determination of the prokaryotic biomass, prokaryotic cell size was converted into bio-volume following inter-calibration with scanning electron microscope (SEM) based size determinations and converted into carbon content assuming 310 fgC μm^–3^ (Danovaro, 2010), according with previous studies and thus enabling comparisons with available data for the deep seafloor (Danovaro et al., 2016; Rastelli et al., 2016 and reference therein). Data of prokaryotic abundance and biomass were expressed as number of cells and μg C per gram of dry weight sediment, respectively.

### Extracellular Enzymatic Activities

Enzymatic activities were determined for aminopeptidase, β-glucosidase, and alkaline phosphatase by the analysis of the cleavage rates of their artificial fluorogenic substrates: L-leucine-4-methylcoumarinyl-7-amide (Leu-MCA); 4-methylumbelliferyl (MUF)-b-D-glucopyranoside (Glu-MUF), and 4-MUF-P-phosphate (MUF-P), respectively (all from Sigma Chemicals) under saturating substrate concentrations (Danovaro, 2010). Briefly, sediment subsamples were diluted with 0.02-μm pre-filtered sterilized seawater and incubated with above mentioned fluorogenic substrates in the dark at the *in situ* temperature for 1–2 h. The fluorescence of the samples was measured fluorometrically at 380 nm excitation, 440 nm emission (for Leu-MCA) and 365 nm excitation, 455 nm emission (for Glu-MUF and MUF-P), immediately after the addition of the substrate and after the incubation, checking for linearity of the increase in fluorescence during the incubation period (Danovaro, 2010). The fluorescence was then converted into enzymatic activity based on standard curves obtained using standard 7-amino-4-methylcoumarin (Sigma Chemicals) for Leu-MCA and 4-methylumbelliferone (Sigma Chemicals) for both Glu-MUF and MUF-P, and pre-filtered sterilized seawater. The amount of the artificial fluorogenic substrate hydrolyzed by proteases and glucosidases were converted respectively into protein and carbohydrate degradation rates using 72 mg of C per micromole of substrate hydrolyzed. Enzymatic activities were expressed as nmol of substrates hydrolyzed in 1 h and normalized to sediment dry weight. Cell-specific enzymatic activity was calculated as the ratio between enzymatic activities and prokaryotic abundances and expressed as nmol of substrate hydrolyzed in 1 h per cell.

### Viral Abundance

According to Danovaro (2010), the sediment samples were sonicated three times (Branson Sonifier 2200, 60 W) for 1 min after addition of 0.02 μm pre-filtered tetrasodium pyrophosphate solution (final, 5 mM). In order to eliminate uncertainties in virus counting due to extracellular DNA interference, sub-samples were supplemented with DNase I from bovine pancreas (5 U mL^–1^ final concentration) and incubated for 15 min at room temperature. The samples were properly diluted with 0.02-μm pre-filtered seawater, filtered onto 0.02-μm-pore-size Al_2_O_3_ filters (Anodisc; diameter 25 mm) and then stained with 100 μl of SYBR Gold 2x (diluting stock solution with 0.02-μm pre-filtered TE buffer: 10 mM Tris-HCl, 1 mM EDTA (Shibata et al., 2006). Filters were incubated in the dark for 20 min, rinsed three times with 3 ml of 0.02-μm pre-filtered Milli-Q water, dried under laminar flow hood and then mounted on glass slides with 20 μl of antifade solution (50% phosphate buffer pH 7.8, 50% glycerol, 0.5% ascorbic acid). The abundance of viral particles counts was obtained by EFM under blue light (Zeiss Axioskop 2MOT, magnification: 1,000×; excitation BP 450–490 nm) examining at least 20 fields per slide, and at least 400 viral particles per filter. Viral abundance was expressed as number of viral particles per gram of dry weight sediment.

### Viral Production and Turnover, Virus-Induced Prokaryotic Mortality (VIPM), and Contribution of Viral Lysis to the Microbial C Cycling

Viral production was determined by using the dilution technique (i.e., a dilution-based procedure) by measuring the increase in viral abundance in the interval between the beginning of the experiment and 6–12 h later (Dell’Anno et al., 2009, 2015). Samples from the top 1 cm of undisturbed deep-sea sediments were diluted immediately in sterile tubes (50 ml) after retrieval with virus-free seawater (0.02 μm pre-filtered), collected at the sediment-water interface of each benthic site (Danovaro et al., 2016; Rastelli et al., 2016). A standard dilution of sediment samples with virus-free seawater was used (sediment to virus-free seawater 1:10 vol:vol). Replicate samples (*n* = 3) for viral counts were collected immediately after dilution of the sediments in sterile tubes (15 ml) and after 3, 6, and 12 h of incubation in the dark at *in situ* temperature. Subsamples were then analyzed as reported above for the determination of viral abundance. The viral production was assessed as the maximum increment of viral abundance observed during the incubations, divided by the incubation time-lapse (in hours) (Dell’Anno et al., 2009).

The number of prokaryotes killed by viruses was estimated from the ratio between viral production and burst size previously determined for hadal sediments (values of 35 and 45; Danovaro et al., 2016). The virus-induced prokaryotic mortality (VIPM) was estimated as the ratio between the number of prokaryotes killed by viruses and the total number of prokaryotes present in the samples and expressed as percentage. Viral turnover was calculated as the ratio between viral production and abundance and expressed as turnover rate (h^–1^).

The concentration of organic carbon (C) released from the cells lysed by viruses in each sediment sample (expressed as mg C m^–2^ d^–1^ considering a sediment density of 1.8 and 50% of water content) was calculated by multiplying the cell size for the number of cells killed per hour (i.e., calculated as the ratio between viral production and burst size).

### Statistical Analysis

For each of the variable analyzed in this study, univariate distance-based permutational analyses of variance (PERMANOVA) was applied to assess differences between sampling stations (six levels). The analyses were followed by the pair-wise test when significant differences were encountered (*p* < 0.05). The analyses were carried out on Euclidean distances using 999 permutations with Monte Carlo simulation, considering all the factors as fixed and unrestricted permutation of raw data. Each statistical analysis was carried out by using the PRIMER v.6.1.12 & PERMANOVA + v.1.0.2 software.

## Results

### Biochemical Composition of Organic Matter and Photosynthetic Pigments

The results of the biochemical composition of organic matter in the surface sediments are shown in Supplementary Figure S1. Protein concentrations ranged from 0.19 ± 0.08 to 9.51 ± 0.77 mg g^–1^ of dry sediment, respectively in the abyssal site surrounding the Mariana Trench and in the bottom site of the Japan Trench. Carbohydrate concentrations ranged from 1.02 ± 0.22 to 4.60 ± 0.33 mg g^–1^ (in the abyssal sites surrounding the Mariana and Japan trenches, respectively), while lipid concentrations from 0.11 ± 0.03 to 2.48 ± 0.55 mg g^–1^ (in the abyssal site neighboring Izu-Ogasawara Trench and in the bottom site of the Japan Trench, respectively).

The Japan Trench sediments contained significantly higher concentrations of proteins and lipids (*p* < 0.01) when compared with data reported from the Izu-Ogasawara and Mariana trenches. The sediments of the Izu-Ogasawara Trench were characterized by the highest carbohydrate concentrations (2.63 ± 0.03 mg g^–1^) among all of the trenches investigated in the present study. Both protein and lipid concentrations in the bottom sediments from the three trenches were significantly higher than those from their related abyssal sites. Instead, carbohydrates concentration in the Japan Trench site was significantly lower than in the abyssal plain site (2.37 ± 0.2 and 4.59 ± 0.3 mg g^–1^ respectively; *p* < 0.01), whereas no differences were observed between the Mariana Trench site and its related abyssal site. The Izu-Ogasawara Trench site, instead, showed significantly higher carbohydrates concentrations compared to its relative abyssal site (*p* < 0.01).

Biopolymeric carbon contents (Supplementary Figure S2A) ranged from 0.56 ± 0.14 to 7.47 ± 0.82 mg g^–1^ with values significantly different among the benthic sites of the investigated trenches (the Japan Trench exceeding 3 and 5 times the concentrations that were present in the Izu-Ogasawara and Mariana trenches, respectively), being higher in hadal systems compared to their related abyssal sites. The concentrations of total phytopigments were significantly higher in the benthic site of the Japan Trench (269.32 ± 13.81 μg g^–1^) than in both the Mariana Trench (6.83 ± 0.25 μg g^–1^) and the related abyssal site (84.09 ± 11.43 μg g^–1^; Supplementary Figure S2B) (both *p* < 0.01).

### Prokaryotic Abundance, Cell Size, and Biomass

**FIGURE 2:**
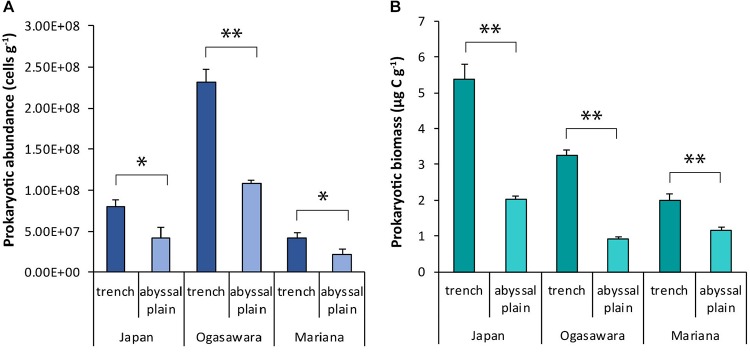
**(A)** Prokaryotic abundance and **(B)** prokaryotic biomass. Reported are the mean values and related SDs for each hadal trench and related abyssal site. Statistical significance is tested between hadal trench sites and abyssal sites. *^∗^p* < 0.05, *^∗∗^p* < 0.01.

Overall, the prokaryotic abundances were significantly higher (*p* < 0.05 and *p* < 0.01) within the trench sites than in their related abyssal sites, with the highest values in the benthic sites from the Izu-Ogasawara Trench (2.31 ± 0.17 × 10^8^ cells sediment g^–1^), followed by those within the Japan and the Mariana trenches (8.02 ± 0.8 × 10^7^ and 4.16 ± 0.7 × 10^7^ cells g^–1^, respectively; Figure 2A).

Prokaryotic cell size ranged from 0.03 ± 0.00 to 0.22 ± 0.01 μm^3^ per cell, in the abyssal site surrounding the Izu-Ogasawara Trench and in the site inside the Japan Trench (Supplementary Figure S3). No significant differences were observed between hadal and abyssal sites, with the exception of the Izu-Ogasawara Trench and its related abyssal (0.05 ± 0.001 and 0.03 ± 0.00 respectively μm^3^ cell^–1^). Prokaryotic abundance and cell size in the sediments of the three hadal trenches investigated were negatively related (Y = −1 × 10^9^X + 0.3 × 10^9^, *R* = 0.85, *n* = 6, *p* < 0.01). Prokaryotic biomasses followed a pattern similar to that of the abundances, with values significantly higher (*p* < 0.01) within the trench sites compared to their abyssal sites. However, the highest value of prokaryotic biomass was found in the benthic site of the Japan Trench (5.39 ± 0.4 μg C g^–1^), followed by those from Izu-Ogasawara and Mariana trenches (3.3 ± 0.13 and 2.01 ± 0.17 μg C g^–1^; Figure 2B).

### Extracellular Enzymatic Activity and Degradation Efficiency

**FIGURE 3:**
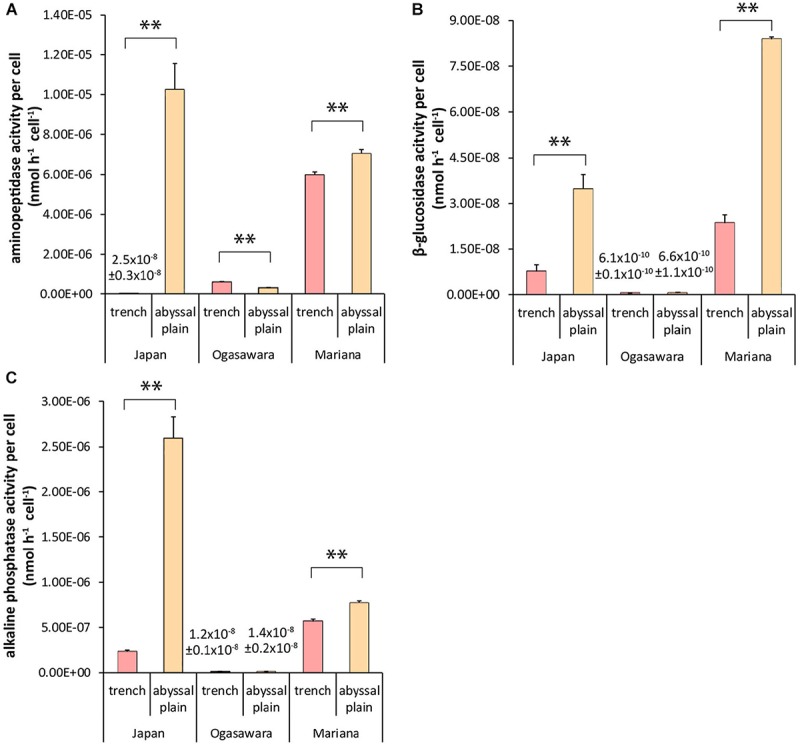
Extracellular enzymatic activities per prokaryotic cell: **(A)** specific aminopeptidase activity, **(B)** specific β-glucosidase activity, and **(C)** specific alkaline phosphatase activity. Reported are the mean values and related SDs for each hadal trench site and related abyssal site. Statistical significance is tested between hadal trenches and abyssal sites. *^∗∗^p* < 0.01.

Aminopeptidase activity ranged from 2.0 ± 0.23 to 433.2 ± 54.7 nmol h^–1^ g^–1^ (in the bottom site of the Japan Trench and in its control abyssal site, respectively), while alkaline-phosphatase and β-glucosidase activity from 1.50 ± 0.19 to 109.6 ± 10.1 nmol h^–1^ g^–1^ and from 0.02 ± 0.01 to 0.89 ± 0.15 nmol h^–1^ g^–1^ in the abyssal sites surrounding the Izu-Ogasawara and Japan trenches (Supplementary Figures S4A–C). The Mariana Trench presented significantly higher values for each enzymatic activity compared to the other two trenches (*p* < 0.01), with the exception of the β-glucosidase activity that exceeded only the value found in the Izu-Ogasawara Trench (0.6 ± 0.04 and 0.05 ± 0.01 nmol h^–1^ g^–1^ respectively, *p* < 0.01). The aminopeptidase activity normalized per prokaryotic cell (used here as a proxy of degradation efficiency) ranged from 2.49 ± 0.29 × 10^–8^ to 1.02 ± 0.13 × 10^–5^ nmol h^–1^ cell^–1^ in the Japan Trench and its control site, the β-glucosidase activity per cell between 6.1 ± 0.1 × 10^–10^ and 8.39 ± 0.06 × 10^–8^ nmol h^–1^ cell^–1^ in the Izu-Ogasawara Trench and the abyssal site related with the Mariana Trench, and the alkaline phosphatase activity per prokaryotic cell ranged between 1.23 ± 0.13 × 10^–8^ and 2.59 ± 0.24 × 10^–6^ nmol h^–1^ cell^–1^ in the site of Izu-Ogasawara Trench and in the abyssal site related with the Japan Trench (Figures 3A–C). The specific enzymatic activities were always significantly lower in the sites of the Mariana and Japan trenches than in the related abyssal plain sediments (*p* < 0.01). In the site of the Izu-Ogasawara Trench, the aminopeptidase per prokaryotic cell was significantly higher than in the surrounding abyssal site (*p* < 0.01), whereas alkaline phosphatase and β-glucosidase activities per prokaryotic cell were not significantly different compared to the abyssal values.

### Viral Abundance and Virus to Prokaryote Ratio

**FIGURE 4:**
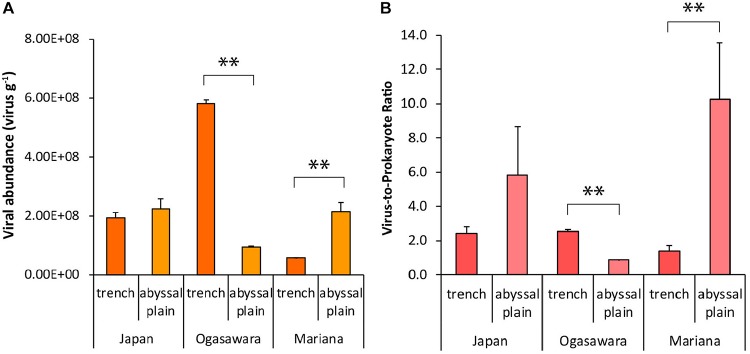
**(A)** Viral abundance (particles g^–1^ of dry weight sediment) and **(B)** virus-to-prokaryote ratio. Reported are the mean values and related SDs for each hadal trench site and related abyssal site. Statistical significance is tested between hadal trench sediments and abyssal sites. *^∗∗^p* < 0.01.

Viral abundances ranged from 5.7 ± 0.23 × 10^7^ viral particles g^–1^ to 5.8 ± 0.11 × 10^8^ viral particles g^–1^ in the bottom sites of the Mariana and Izu-Ogasawara trenches, respectively, and did not show a clear spatial pattern (Figure 4A). No significant differences were found between viral abundances in the hadal sites of the Japan Trench and its related abyssal site, whereas viral abundances were significantly higher in the benthic site of the Izu-Ogasawara Trench than in the adjacent abyssal sites (*p* < 0.01). Conversely, the benthic site of the Mariana Trench showed significantly lower viral abundances than in the abyssal sites (*p* < 0.01). The virus to prokaryote abundance ratio (VPR) ranged from 0.9 ± 0.0 to 10.3 ± 3.3 (in the abyssal sites surrounding the Izu-Ogasawara and Mariana trenches, respectively; Figure 4B). In the Mariana Trench this ratio was significantly lower than in the abyssal sites whereas in the Izu-Ogasawara Trench site it was significantly higher (*p* < 0.01). In the Japan Trench site, the ratio was not significantly different compared to the VPR found in the surrounding abyssal site, which, however, presented highly variable values (Figure 4B).

### Viral Production and Turnover

**FIGURE 5:**
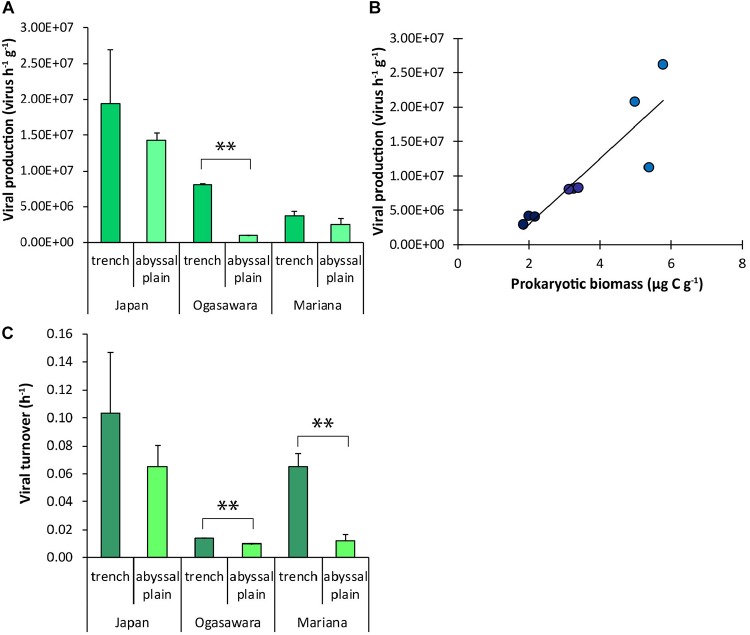
**(A)** Viral production. **(B)** Relationship between prokaryotic biomass and viral production values in each hadal trench. The equation of the fitting line is: Y = 5 × 10^6^ X – 6 × 10^6^; *R* = 0.89; *n* = 9; *p* < 0.01. Dark blue dots: Mariana Trench; blue dots: Izu-Ogasawara Trench; light blue dots: Japan Trench. **(C)** Viral turnover rate. In **(A,C)**, reported are the mean values and related SDs for each hadal trench site and related abyssal site. Statistical significance is tested between hadal trench sediments and abyssal sites. *^∗∗^p* < 0.01.

Benthic viral production ranged from 9.45 ± 0.6 × 10^5^ to 1.94 ± 0.76 × 10^7^ viruses g^–1^ h^–1^ in the abyssal site surrounding the Izu-Ogasawara and within the Japan Trench, respectively (Figure 5A). The rates varied significantly amongst the three trenches, with lowest value found in the Mariana Trench (3.72 ± 0.67 × 10^6^ viruses g^–1^ h^–1^). Overall, viral production was always higher in the sediments from the trench sites than in their adjacent abyssal sites (with significant differences in the Izu-Ogasawara Trench, *p* < 0.01; Figure 5A). Viral production was found significantly related to prokaryotic biomass (Y = 5 × 10^6^X – 6 × 10^6^, *R* = 0.89, *n* = 9, *p* < 0.01; Figure 5B). The viral turnover rate varied from 0.01 ± 0.00 to 0.1 ± 0.04 h^–1^ (in the abyssal site related with the Izu-Ogasawara Trench and in the site from the Japan Trench, respectively) with higher rates within the trench sites, and significantly higher in the benthic sites of the Izu-Ogasawara and Mariana trenches compared to the related abyssal sites (*p* < 0.01, Figure 5C).

### Virus-Induced Prokaryotic Mortality and C Released Due to Viral Lysis

**FIGURE 6:**
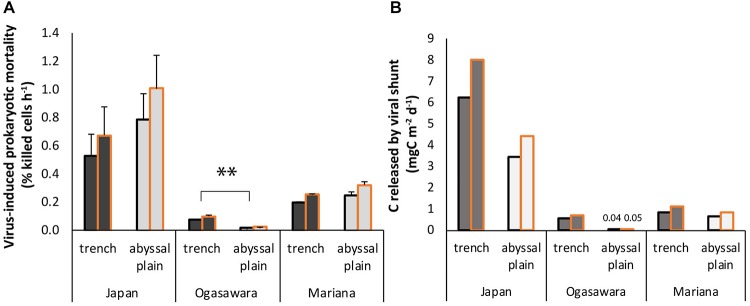
**(A)** Virus-induced prokaryotic mortality (VIPM). Reported are the mean values using two different burst sizes (BS), 45 (black contour) and 35 (gold contour), respectively (Danovaro et al., 2008b, 2016), and related SDs for each hadal trench site and related abyssal site. Statistical significance is tested between hadal trench and abyssal sites. ^∗^*^∗^p* < 0.01. **(B)** C released by killed prokaryotes due to viral lysis. Reported are the value per each hadal trench site and related abyssal sites obtained by using two different burst sizes (BS), 45 (black contour), and 35 (gold contour), respectively.

Virus-induced prokaryotic mortality, obtained by the number of killed cells h^–1^ (using BS values of 45 and 35, Supplementary Figure S5) and total prokaryotic abundance, showed the highest values in the bottom site of the Japan Trench among all the hadal sites (0.55–0.70% h^–1^ compared to 0.08–0.10% h^–1^ in the Izu-Ogasawara Trench and 0.20–0.26% h^–1^ in the Mariana Trench; Figure 6A). Only in the Izu-Ogasawara Trench, VIPM was significantly higher (*p* < 0.01) than in the respective abyssal site (0.02 and 0.08% h^–1^ respectively; Figure 6A), while the values from the other two trenches did not show significant differences compared to their surrounding abyssal plains. The carbon released by the viral lysis of the prokaryotes corresponded to 6.3–8.0 mg C m^–2^ d^–1^ within the Japan Trench (Figure 6B). In the other trench sites, it ranged from 0.55–0.71 to 0.86–1.11 mg C m^–2^ d^–1^ (in the Izu-Ogasawara and Mariana trenches, respectively). Overall, the C released from prokaryotes lysed by viruses was higher within the hadal sites of the trenches than in their related abyssal sites.

## Discussion

Prokaryotic abundances in the surface sediments of the investigated trench sites are within the ranges of values reported for deep-sea sediments worldwide (10^7^–10^9^ cells g of sediment dry weight, Danovaro et al., 2008b, 2016) and are comparable to values previously observed in the Mariana and Atacama trenches (Danovaro et al., 2003; Glud et al., 2013). Prokaryotic abundances in the sediments of the three trenches were significantly higher than in the respective abyssal sites suggesting that hadal sediments can support the growth of thriving prokaryotic assemblages. This is consistent with previous findings from the Izu-Ogasawara Trench, which showed prokaryotic abundances and O_2_ consumption rates in the hadal sediments exceeding those from surrounding abyssal sites (Wenzhöfer et al., 2016). A similar pattern was also reported in the sediments of the Challenger Deep, in the Mariana Trench and its reference abyssal site (Glud et al., 2013).

Prokaryotic abundance and cell size in the sediments of the investigated hadal trenches were negatively related. In particular, in the Izu-Ogasawara Trench, we observed the highest prokaryotic abundances and the lowest cell sizes. The reason why in the hadal sediments of Izu-Ogasawara Trench, and in the surrounding abyssal site, the cell size was so small when compared to other investigated sites is unclear. However, it is possible to hypothesize that this feature is associated with the specific taxonomic composition of the microbial assemblages of the hadal trench and geographical area.

The values of benthic prokaryotic biomass obtained by combining data of cell size and abundance were significantly higher in the hadal trenches than in the surrounding abyssal sites, suggesting that these ecosystems are microbial hot spots. This was also confirmed by the high values of enzymatic activities in the hadal trench sediments, typically higher than those observed at abyssal depths. The only exception was the Japan Trench site, where both enzymatic activities and efficiency (determined as ratio between enzymatic activity and prokaryotic abundance) were lower than in the surrounding abyssal site. Since prokaryotic biomasses and biopolymeric C concentrations were high in the trench, with protein concentrations even higher than in benthic coastal sites (Pusceddu et al., 2009), the reason for the enzymatic degradation pattern remains unclear. A similar prokaryotic efficiency was also observed in the sediments of Mariana and Izu-Ogasawara trenches. The discrepancy between low efficiency in organic matter degradation despite the availability of trophic resources could be due to an inhibition of extracellular enzymatic activities due to high concentrations of organic substrates (i.e., proteins) or the presence of refractory compounds (Boetius and Lochte, 1994; Arnosti and Holmer, 2003). This phenomenon has been repeatedly reported also in deep-sea sediments (Fabiano and Danovaro, 1998) and could influence the prokaryotic degradation efficiency of organic matter. In addition, a non-heterotrophic pathway of biomass production, such as mixotrophy and/or chemolitotrophy, could contribute to support the high abundances and biomasses of prokaryotes, as previous studies suggested that archaea represent a relevant prokaryotic component in the surface sediments of hadal trenches (Nunoura et al., 2018; Zhang et al., 2018).

Viral abundances in the surface sediments of the investigated trenches were in the range of previously published values from hadal sediments (Yoshida et al., 2013) and other deep-sea sediments worldwide (Danovaro et al., 2008b). Such abundances were generally similar or higher than the values obtained in deep-sea sub-surface sediments (Corinaldesi et al., 2010; Middelboe et al., 2011; Engelhardt et al., 2013; Yanagawa et al., 2014), possibly depending on the availability of host abundances to be infected or other factors (e.g., viral decay rates, preservation mechanisms of viruses in subsurface layers, Corinaldesi et al., 2012; Cai et al., 2019).

Viral abundances and VPR in the hadal and abyssal sites investigated did not showed a clear pattern of distribution. However, benthic viral abundance in the Izu-Ogasawara Trench was higher than in the other trenches, and only in this site VPR exceeded the value found in the respective abyssal site. VPR values have been used in previous studies to infer relationships between viruses and hosts (Engelhardt et al., 2014; Parikka et al., 2017). However, the VPR is a numerical value that merely expresses the relative importance of viruses over their hosts at a specific site and time (Corinaldesi et al., 2010; Parikka et al., 2017). Therefore, a better assessment of the virus–prokaryote interactions can be obtained by determining directly the viral production and VIPM rates (Corinaldesi et al., 2019), as in the present study.

Viral production in the hadal trench sediments was similar or even higher than in the respective abyssal sites. However, overall viral production rates were comparable to the values already obtained from other benthic deep-sea surface sediments worldwide (Danovaro et al., 2008b). These findings are not the result of artifacts as previous studies conducted in deep-sea ecosystems revealed that pressure conditions do not affect the measures (Danovaro et al., 2016; Rastelli et al., 2016). In addition, the method utilized, the sediment-dilution approach for determining viral production, is the most widely used technique in benthic environments (thus enabling a comparison with available data) and has the advantage of minimizing the impact of protozoa and/or fauna grazing on prokaryotes during incubation (Danovaro et al., 2008a; Dell’Anno et al., 2015; Rastelli et al., 2016).

Viruses in the sediments of the hadal trenches showed short turnover times (0.3–2.5 days), especially in the Izu-Ogasawara and Mariana trenches, where these values were 1.4–5.4 times shorter than in the respective abyssal sites and in other previously published deep-sea sediments worldwide (i.e., 2–3 days; Danovaro et al., 2016). In addition, VIPM was high in all the investigated trenches, especially in the Japan Trench (where the values were ca. double than those found in the Mariana Trench and six times higher compared to those obtained in the Izu-Ogasawara Trench). Viral lysis rates obtained in the Japan Trench were even higher than mean values reported for other deep-sea habitats (Danovaro et al., 2016). These estimates were obtained assuming burst size values reported in previous investigations conducted in abyssal-hadal ecosystems, so they should be considered as potential rates.

To be sustainable over time, the rates of virus-induced mortality of prokaryotes should be balanced by cellular turnover. Despite turnover times of prokaryotes were not determined in the present study, there is evidence that prokaryotic replication in benthic deep-sea surface sediments may take from few to some weeks (30 days in surface hadal sediments, Danovaro et al., 2008b, 2016). This suggests that, especially in the hadal trenches, viral lysis is responsible for the abatement of almost the entire new biomass production of benthic prokaryotes, thus representing a key mechanism controlling their turnover rate.

In the present study we found a significant and positive correlation between viral production and prokaryotic biomasses in all trenches. Correlation analyses do not allow inferring any cause-effect relationships; however, our results suggest that a larger mass of prokaryotic standing stock can support higher viral replication rates. And, vice versa, that viral lysis, through the release of labile cellular material available for microbial consumption, might contribute to boost prokaryotic biomass production.

Since previous studies suggested that Archaea represent an important component of the prokaryotic biomass in benthic hadal ecosystems (Nunoura et al., 2018; Zhang et al., 2018) and that the impact of viral infection is higher on Archaea than on Bacteria (Danovaro et al., 2016) it is possible to hypothesize that viral infection represents a key mechanism controlling the turnover of Archaea in hadal trenches.

In the Japan Trench, viral lysis caused potentially the release of ca. 6–8 mg C m^–2^ d^–1^. This C amount was higher than the values obtained from the other hadal and abyssal sites in this study, as well as than values previously reported for other benthic deep-sea sediments worldwide (Corinaldesi et al., 2012; Danovaro et al., 2016). The large release of labile matter due to viral lysis can stimulate heterotrophic activity and organic matter mineralization rates (Guenet et al., 2010; Schmidt et al., 2011; Rastelli et al., 2017). However, in the hadal trenches (and especially in the Japan Trench), the high rates of viral production were associated with a low organic matter degradation efficiency. These patterns suggest that the large availability of trophic sources could reduce the need for prokaryotes of enzymatic digestion of the organic material to obtain the sources of organic C, N, and P especially in the organic rich hadal sediments, such as Japan Trench. Therefore, our findings suggest that prokaryotic biomass can be fueled through the recycling of labile material made available by viral lysis. Such a stimulatory effect mediated by viruses has been previously documented not only toward the microbial heterotrophic metabolism but also chemoautotrophic metabolism (Rastelli et al., 2016).

Since the C release by viral lysis changes from trench to trench, the specific environmental conditions (including the different availability of trophic sources), are expected to play a key role in microbial dynamics of trench sediments. The combined effect of the environmental factors needs to be further investigated to better understand the functioning of microbial food-webs and their implications on biogeochemical cycles in these extreme ecosystems.

## Data Availability

The raw data supporting the conclusions of this manuscript will be made available by the authors, without undue reservation, to any qualified researcher.

## Author Contributions

CC, AD, and RD conceived the study. ER provided samples for the study. EM, ER, and MT performed the laboratory analyses. EM, ER, MT, AD, and CC contributed to the data elaboration and interpretation. TN and HN allowed the oceanographic cruise and samples collection. EM, CC, and AD wrote the manuscript and all the authors contributed to the discussion and its finalization.

## Conflict of Interest Statement

The authors declare that the research was conducted in the absence of any commercial or financial relationships that could be construed as a potential conflict of interest.
