# Supplementary material for: Viral Infections Boost Prokaryotic Biomass Production and Organic C Cycling in Hadal Trench Sediments
Source: Front Microbiol. 2019 Aug 23;10:1952. doi: 10.3389/fmicb.2019.01952 (PMC6716271; doi:10.3389/fmicb.2019.01952)
Supplement: Supplementary file 1 [file Data_Sheet_1.docx]

Supplementary Material

# Supplementary Figures and Tables


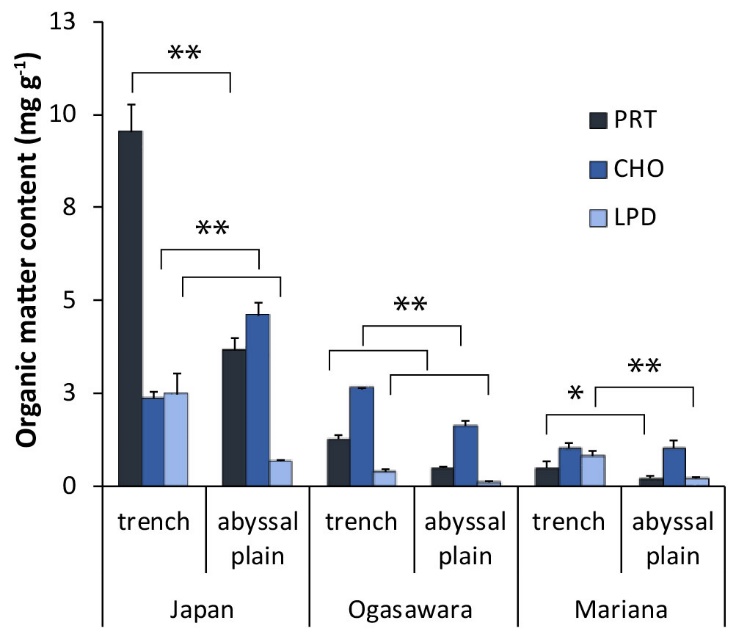


**Supplementary Figure S1.** Organic matter contents in terms of proteins (PRT), carbohydrates (CHO) and lipids (LPD) of the surface sediments. Reported are the mean values and related SDs for each hadal trench and related abyssal site. Statistical significance is tested between hadal trenches and abyssal sites. **p* < 0.05, ***p* < 0.01


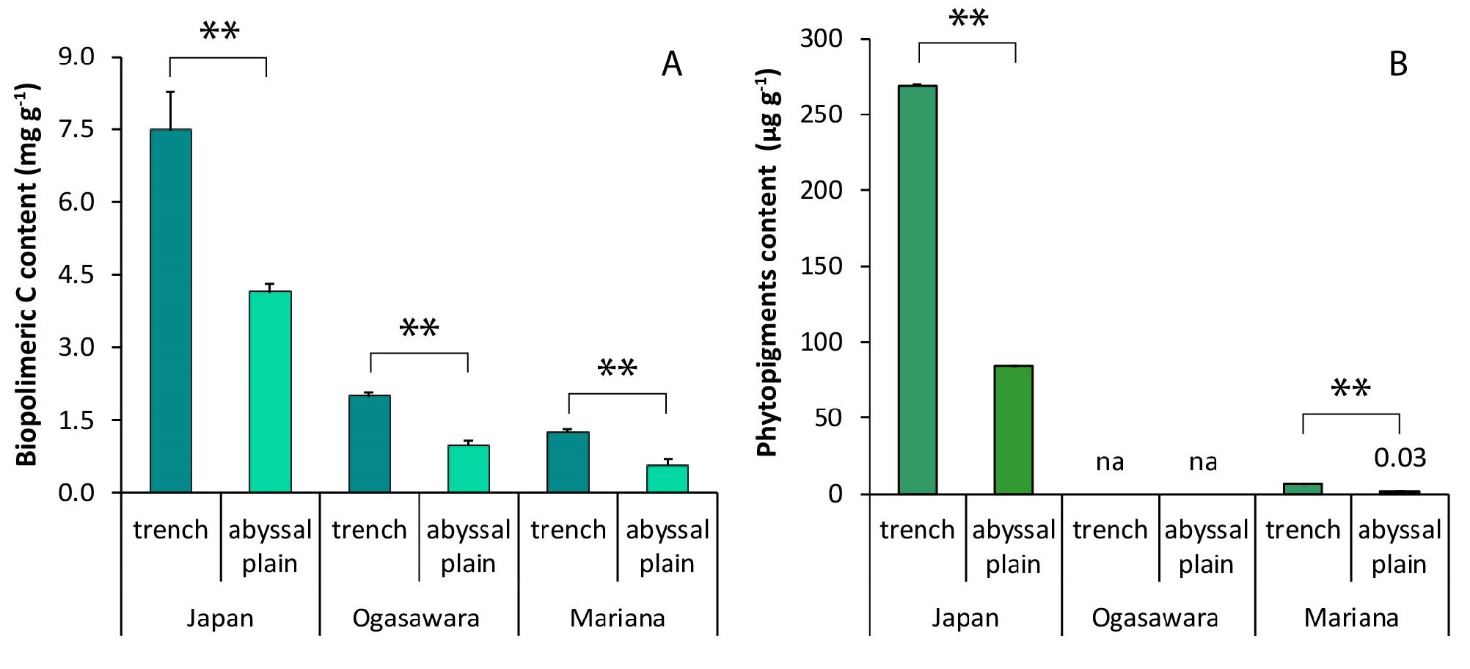


**Supplementary Figure S2 (A)** Biopolimeric carbon content, and **(B)** total phytopigments content of the surface sediments. Reported are the mean values and related SDs for each hadal trench and related abyssal site. Statistical significance is tested between hadal trenches and abyssal sites. ***p* < 0.01


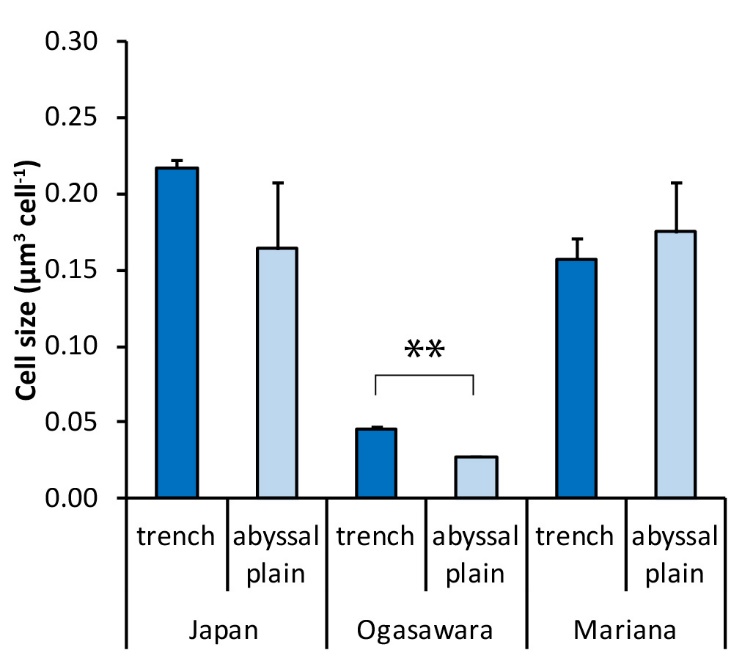


**Supplementary Figure S3.** Prokaryotic cell size of the surface sediments. Reported are the mean values and related SDs for each hadal trench and related abyssal site. Statistical significance is tested between hadal trenches and abyssal sites. ***p* < 0.01


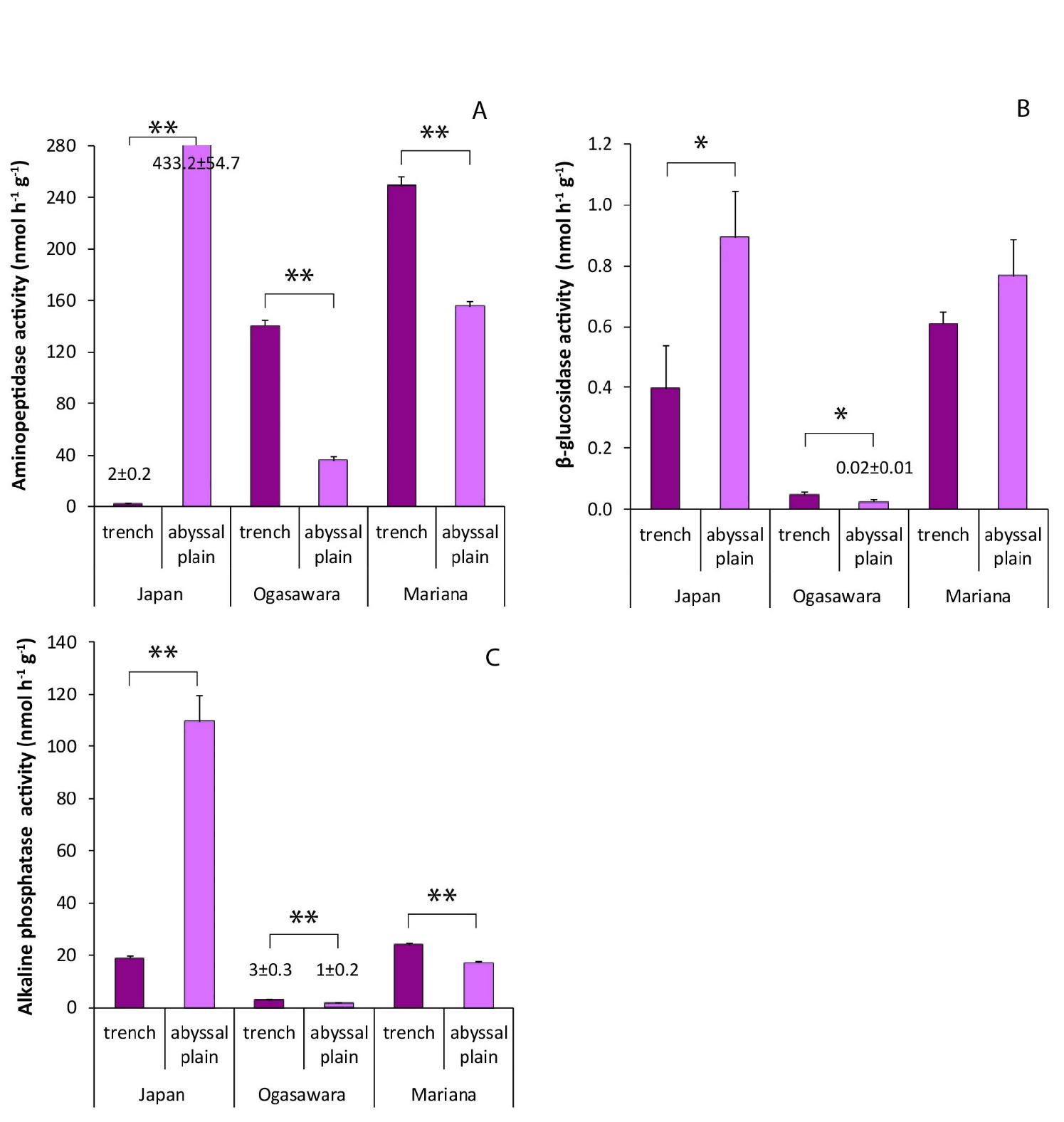


**Supplementary Figure S4.** Measures of extracellular enzymatic activities: **(A)** aminopeptidase, **(B)** β-glucosidase, and **(C)** alkaline phosphatase examined using surface sediments. Reported are the mean values and related SDs for each hadal trench and related abyssal site. Statistical significance is tested between hadal trenches and abyssal sites. **p* < 0.05, ***p* < 0.01


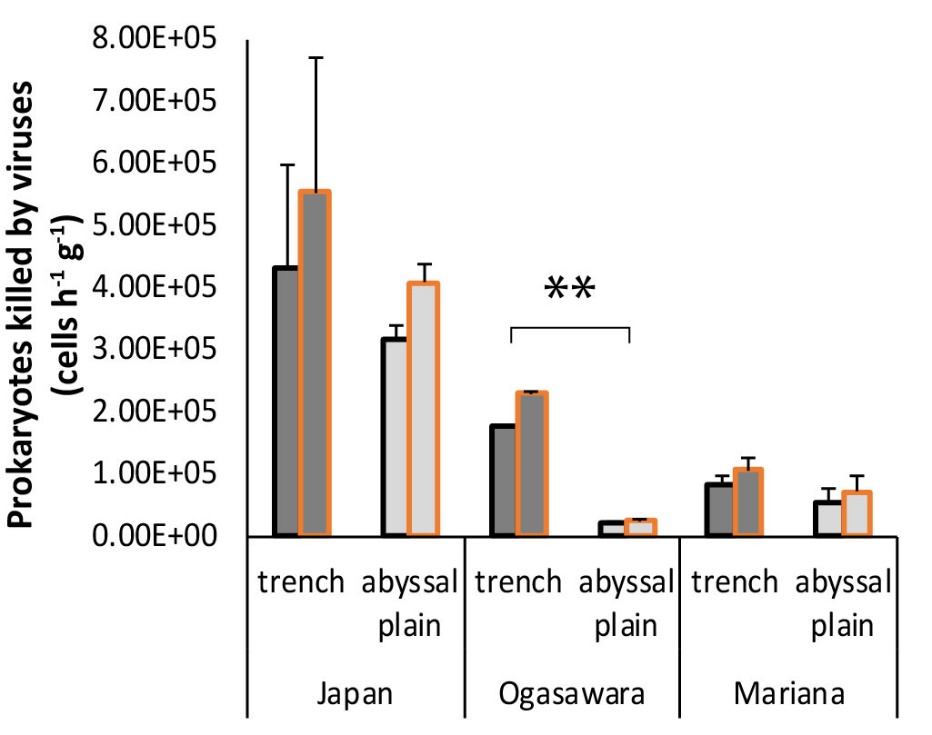


**Supplementary Figure S5.** Prokaryotic cells killed by viral lysis in the surface sediments calculated by adopting two different burst sizes (BS), 45 (black contour) and 35 (gold contour), respectively (Danovaro et al., 2016). Reported are the mean values and related SDs for each hadal trench and related abyssal site. Statistical significance is tested between hadal trenches and abyssal sites. ***p* < 0.01

**References**

1. Danovaro, R., Dell’Anno, A., Corinaldesi, C., Rastelli, E., Cavicchioli, R., Krupovic, M., et al. (2016). Virus-mediated archaeal hecatomb in the deep seafloor. *Sci. Adv*. 2, e1600492. DOI: 10.1126/sciadv.1600492
